# Modified lateral rhinotomy for fronto-ethmoid schwannoma in a child: a case report

**DOI:** 10.1186/1757-1626-3-64

**Published:** 2010-02-18

**Authors:** Anurag Ramavat, Rakesh Kumar, C Venkatakarthikeyan, Ayushi Jain, Ramesh C Deka

**Affiliations:** 1 Department of Otorhinolaryngology & Head & Neck Surgery, All India Institute of Medical Sciences, Ansari Nagar, New Delhi-110029, India; 2 Department of Pathology, All India Institute of Medical Sciences, Ansari Nagar, New Delhi-110029, India

## Abstract

Schwannoma of frontoethmoid region is a rare presentation. We report a case of 11-year-old girl with a swelling at the root of nose and nasal dorsum. Based on clinical picture and radiological findings it was not possible to establish a definitive diagnosis. But the histopathological picture was suggestive of schwannoma. A novel surgical approach was adopted to facilitate complete removal of the tumor and provide best possible cosmetic results.

## Introduction

Schwannoma of the frontoethmoid region is a rare entity. 25% to 45% of all schwannomas have been observed in the head and neck region. Out of all head and neck schwanomas, only 4% are seen in the nose and paranasal sinuses (Shuger et al 1981) [1,2]. There are always debate and arguments about the surgical approach and cosmesis for a large mass situated in frontoethmoid area. We used a modified incision to facilitate good exposure with minimum visible scar to excise the mass.

## Case presentation

An 11-year-old girl from North-eastern part of India presented with 2 year history of progressive painless swelling at the root of nose. She had left sided nasal obstruction for one year and had occasional blood stained nasal discharge. Her vision was normal and had normal eye movements. There were no skin lesions/pigmentation over her any body part and had no signs of focal neurological deficits. Local examination revealed a smooth 4 × 3 cm swelling at the root of nose and bony nasal dorsum causing nasal deformity and telecanthus. The skin over swelling was normal and on palpation it was firm, non- tender and non compressible. The nasal bones appeared to be splayed and thinned out in the middle through which tumor was palpable. Anterior rhinoscopy revealed a pinkish mucosa covered mass filling the upper half of left nasal cavity and nasal septum was pushed to the opposite side.

**Figure 1:**
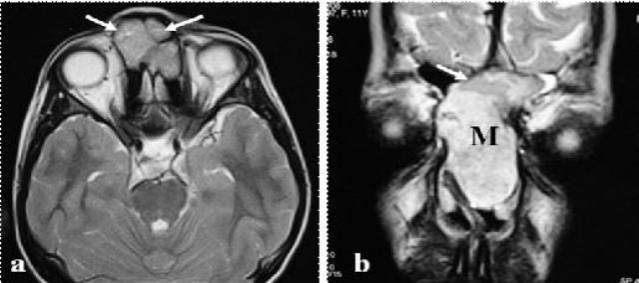
**T2 weighted axial image (a) showing a hyperintense soft tissue mass (arrow) in the nasal cavity anteriorly**. Coronal T2 weighted image showing the mass (M) in the nasal cavity extending into the left frontal sinus (arrow).

Contrast enhanced high resolution computed tomography (HRCT) of the paranasal sinuses revealed soft tissue expansile lesion of 42 × 36 × 20 mm size in left nasal cavity, left frontal sinus and bilateral anterior ethmoid area causing widening of nasal cavity and leading to thinning and erosion of right nasal bone. The mass was reaching upto but not eroding the cribriform plate. There were scattered areas of increased attenuation and mild post-contrast enhancement. Magnetic resonance imaging (MRI) showed a mass lesion of intermediate intensity on T1-weighted images and the T2-weighted signal without the cystic characteristics of the lesion (Figure 1). Based on clinical and radiological findings a differential diagnosis of dermoid, mucocoele or rhabdomyosarcoma was made. Fine needle aspiration cytology showed only blood. Endoscopic surgery under general anaesthesia was planned for tissue biopsy and if found cystic, marsupalisation would be done. On incision it was a solid encapsulated greyish mass. Tissue was sent for histopathological examination and reported as schwannoma. The patient was taken up for excision of the tumor mass using external approach since such a large tumor was not possible to remove by endoscopic approach.

**Figure 2:**
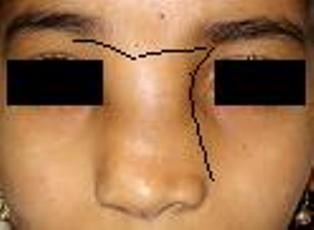
**Preoperative photograph with incision made as depicted by black line**.

**Figure 3:**
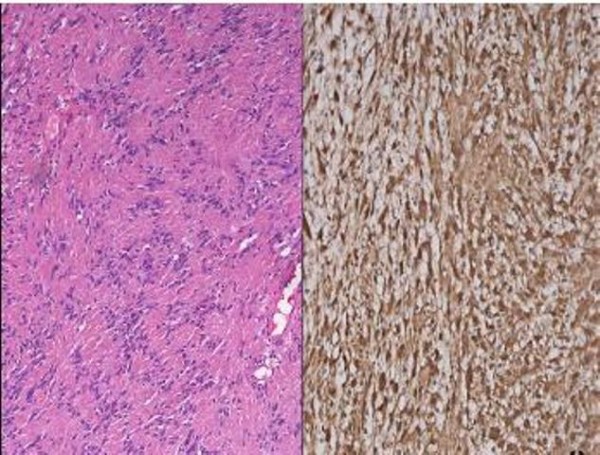
**Photomicrographs demonstrating features typical of a schwannoma comprising of (A) A higher power view showing Verocay body formation consisting of alternating, parallel rows of tumor cell nuclei**. (Hematoxylin and Eosin, × 100); (B) Immunostaining for S-100 protein showing strong nuclear positivity in the fascicles of spindle cells (S-100, × 200)

**Figure 4:**
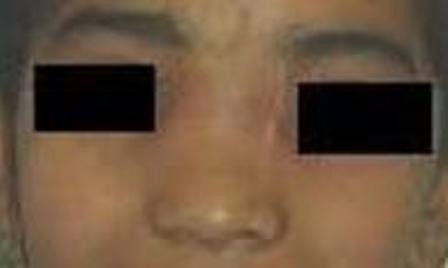
**Postoperative picture after 4 months showing well healed facial scar**.

A left sided modified lateral rhinotomy incision with lateral extension into the eyebrow was made (Figure 2). This was joined superiorly with butterfly incision to have good access. On flap elevation papery thin nasal bones which were deficient in midline seen. The nasal bones were gently reflected off the tumor. A well circumscribed, lobulated mass occupying left frontal sinus, ethmoid sinus and the nasal cavity was seen. The tumor was removed piece meal and was found to be adherent in the area of bony septum. Complete removal of the tumor mass was achieved and confirmed using nasal endoscopy. Intraoperatively bleeding was minimal. Skin flaps along with nasal bone replaced back and sutured after securing hemostasis. Final histopathological and immunohistochemical examination was consistent with sinonasal schwannoma (Figure 4). Postoperative recovery was uneventful and pack was removed on third postoperative day. At 4 months follow-up there is no evidence of disease and skin incision is well healed (Figure 3).

## Discussion

The first description of a schwannoma in the nasal cavity was given from Germany in 1926. The origin of this tumor is from the Schwann cells of the branches of intranasal nerves, ophthalmic and maxillary nerve of the trigeminal nerve and also from the autonomic nervous system. It was difficult to find out the nerve of origin of the tumor in our case but probably it was anterior ethmoidal nerve (due to its site). It can present at any age with no sex or race predisposition and local recurrences are infrequent.

Clinically these tumors present like any other benign mass or polyp and the signs and symptoms depend on the site of tumor and the subsequent involvement of surrounding structures or its pressure effects. The most common symptoms are nasal obstruction, epistaxis, hyposmia and pain. A radiological investigation is essential to know the site and extent of the tumor. The lesions usually have a mottled appearance with peripheral intensification on contrast-enhanced CT scans. MRI is superior to CT scan in differentiating the tumor from inflammatory changes and very useful in evaluating intracranial extension. Sinonasal schwannomas have an intermediate to high intensity on T1-weighted images and the T2-weighted signal depending on the cellularity of the lesion and the cystic characteristics of the lesion [3].

The differential diagnosis includes a spectrum of lesions ranging from angiomas and polyps to malignant tumors, such as melanoma or olfactory neuroblastoma. Since it is an expansile lesion without bone destruction it can be differentiated from malignancy and shows enhancement in CT unlike a mucocele. Electron microscopy and immunohistochemical analysis (S100) is necessary to differentiate a schwannoma from a neurofibroma. Although schwannoma are seen in order of frequency: nasoethmoidal region, maxillary sinus, the nasal cavity, and the sphenoid sinus with only a few reported cases of frontal sinus involvement in conjunction with the ethmoid sinuses [4,5]. Our patient had similar presentation of fronto-ethmoidal sinus involvement. Anterior skull base involvement has been also noted by few authors [6,7].

Complete surgical excision of the tumor should be attempted in first sitting to prevent recurrence. The approach for the resection should be chosen according to the location and extension of the tumor. The aim is to have i) adequate exposure ii) good cosmetic appearance, which is probably the most important from patient's viewpoint. Various combinations of techniques, including lateral rhinotomy, external ethmoidectomy, Caldwell -Luc approach; midface degloving, and/or endonasal endoscopic resection can be used [8,9]. Our case presented with large mass situated at the glabellar region. So single incisions like butterfly incision and Moore's with lateral extension above medial canthus in isolation may not have been adequate. A modified lateral rhinotomy incision was combined with butterfly incision as shown in picture (Figure 2). The advantage of butterfly incision was to have an access to the ethmoid sinuses on both sides and joining it with Moore's incision gave good exposure to the contralateral nasal cavity. So with such a good access the surgery became simple. Further curving the incision gives an advantage during closure by providing landmark for perfect approximation. In addition when scar matures and contracts, the webbing of incision and unsightly scar is avoided. Large Frontoethmoid schwannomas may not be amendable endoscopically so a modified surgical approach may facilitate complete removal of tumor besides giving good scar and better cosmetic appearance.

## Consent

An informed written consent was received from the father (patient being minor) for publication of the manuscript and figures

## Competing interests

The authors declare that they have no competing interests.

## Authors' contributions

AR, data collection, wrote manuscript, assistant surgeon: RK, concept, wrote manuscript, surgeon: CVK, data collection: AJ, histopathological report, microphotograph: RCD, concept, editorial advice

All authors have read and approved the final manuscript.
